# Supplementary figures and images for: Femicide in Mexico: Statistical evidence of an increasing trend
Source: PLoS One. 2023 Dec 22;18(12):e0290165. doi: 10.1371/journal.pone.0290165 (PMC10745190; doi:10.1371/journal.pone.0290165)

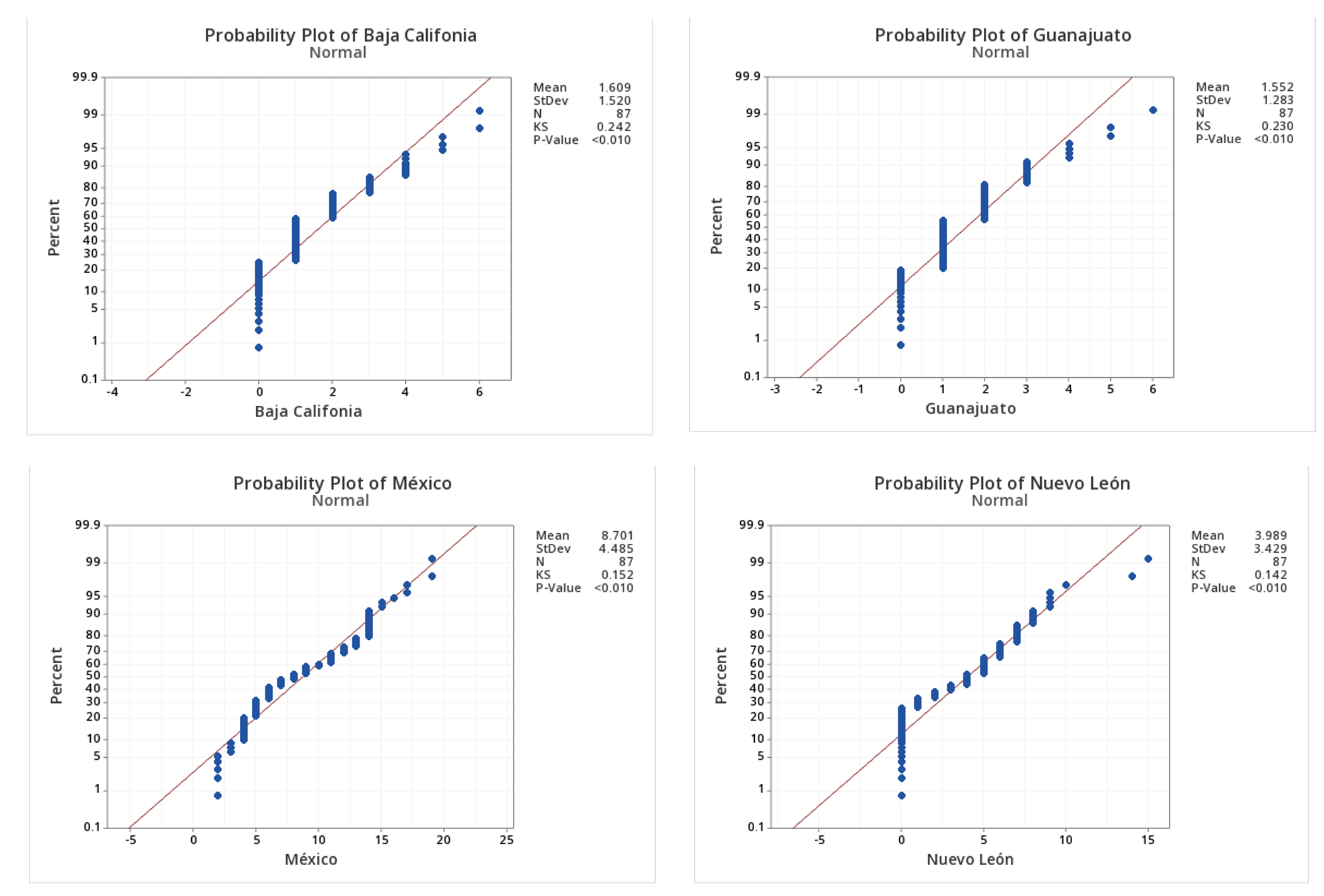

Supplement: S1 Fig — Authors’ elaboration. (TIF) [file pone.0290165.s001.tif]

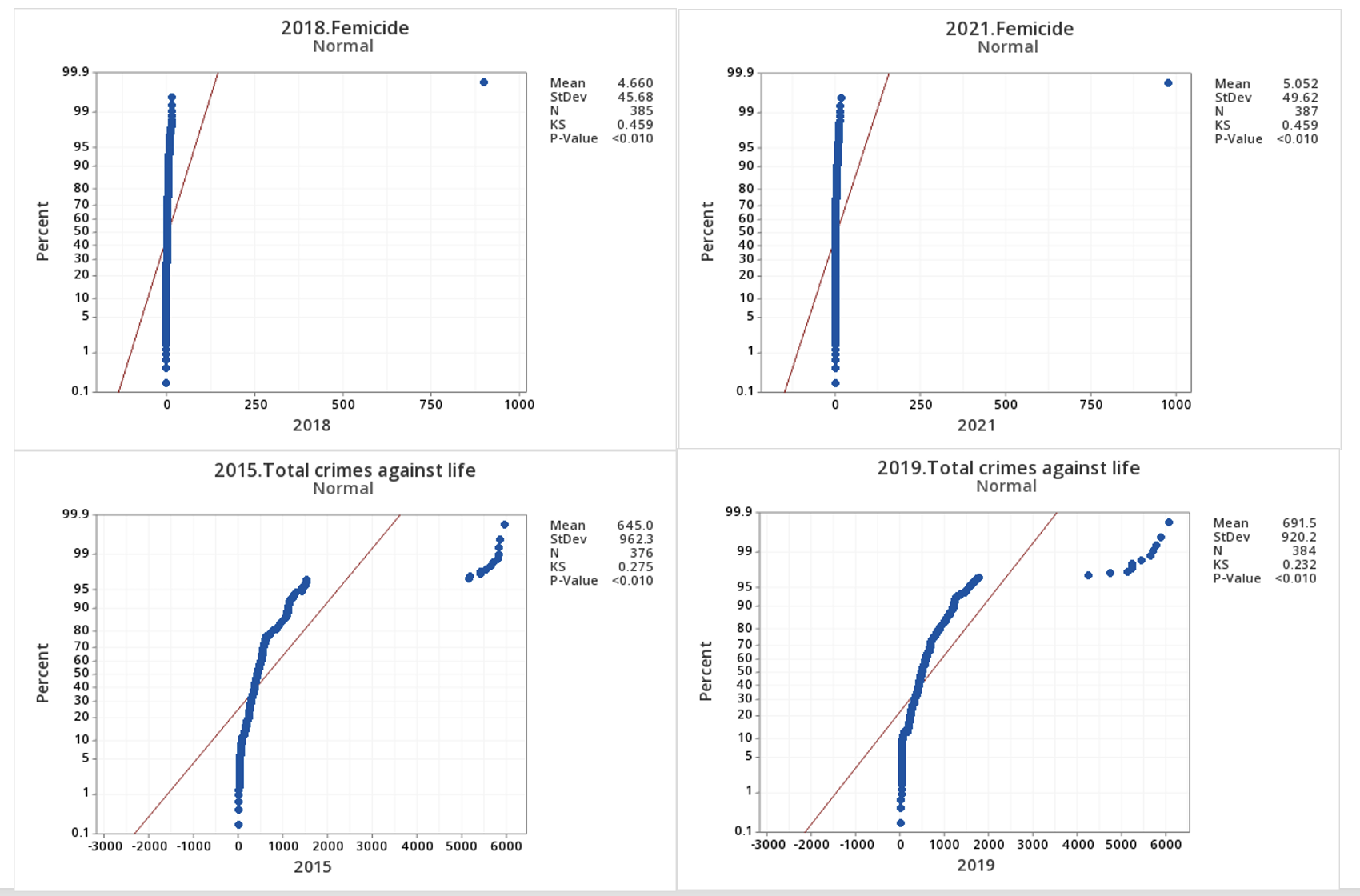

Supplement: S2 Fig — Authors’ elaboration. (TIF) [file pone.0290165.s002.tif]
